# Supplementary material for: Positive association of angiotensin II receptor blockers, not angiotensin-converting enzyme inhibitors, with an increased vulnerability to SARS-CoV-2 infection in patients hospitalized for suspected COVID-19 pneumonia
Source: PLoS One. 2020 Dec 21;15(12):e0244349. doi: 10.1371/journal.pone.0244349 (PMC7751849; doi:10.1371/journal.pone.0244349)
Supplement: S1 Table — (DOC) [file pone.0244349.s001.doc]

**S1 Table**. High Council of Public Health, France. Definition of population with a risk of developing severe COVID-19. March 31, 2020

| People aged ≥ 70 years (even if people aged 50 to 70 years must be monitored more closely) |
| --- |
| Patients with a cardiovascular history of complicated hypertension^a^, stroke, coronary artery disease, cardiac surgery, congestive heart failure NYHA class III or IV |
| Patients with complicated or insufficiently controlled diabetes^b^ |
| Patients with a chronic respiratory disease likely to worsen during a viral infection |
| Patients with chronic kidney disease on dialysis |
| Patients with active or treated cancer (except hormonotherapy) |
| Despite the absence of evidence in the literature, due to an assumed risk of serious COVID-19, taking into account the data known for other respiratory infections^c^, the following are also considered to be at risk of serious COVID-19: |
| - people with congenital or acquired immunosuppression: drug related (anticancer chemotherapy, immunosuppressive treatment, biotherapy and/or corticosteroids at immunosuppressive dose); uncontrolled HIV infection or with CD4 < 200/mm^3^; following a solid organ or hematopoietic stem cell transplant linked to a malignant hemopathy |
| - patients with cirrhosis (at least stage B of the Child Pugh score) |
| - people with obesity (body mass index [BMI] > 40 kg/m^2^), by analogy with influenza A (H1N1), but also obesity with BMI > 30 kg/m^2^ |
| - people with major sickle cell syndrome due to an increased risk of secondary bacterial infection or acute chest syndrome^d^ or with a history of splenectomy |
| - pregnant women in the third trimester of pregnancy, taking into account the available data and considering that they are very limited |

BMI indicates body mass index; COVID-19, coronavirus disease 2019; HIV, human immunodeficiency virus; NYHA, New York Heart Association.

a Cardiac, renal and cerebrovascular complications.

b Unpublished data.

c Obstructive pulmonary disease, severe asthma, pulmonary fibrosis, sleep apnea syndrome, cystic fibrosis in particular.

d Acute chest syndrome is a specific lung disease of sickle cell anemia; it is defined by the association of fever or respiratory symptoms with a pulmonary infiltrate on X-ray.

<https://www.hcsp.fr/Explore.cgi/Telecharger?NomFichier=hcspa20200331_corsarcovprienchadesperrisdeforg.pdf> (accessed May 15, 2020)
