# Supplementary material for: Positive association of angiotensin II receptor blockers, not angiotensin-converting enzyme inhibitors, with an increased vulnerability to SARS-CoV-2 infection in patients hospitalized for suspected COVID-19 pneumonia
Source: PLoS One. 2020 Dec 21;15(12):e0244349. doi: 10.1371/journal.pone.0244349 (PMC7751849; doi:10.1371/journal.pone.0244349)
Supplement: S2 Table — (DOC) [file pone.0244349.s002.doc]

**S2 Table.** **COVHYP study: Causes for exclusion.**

| **Cause** | **Number** |
| --- | --- |
| Death in emergency care unit before RT-PCR and chest imaging | 2 |
| RT-PCR for SARS-CoV-2 not performed | 30 |
| No symptoms of COVID-19 | 43 |
| Second hospitalization for the same patient | 4 |
| Total | 79 |

COVID-19 indicates coronavirus disease 2019; RT-PCR, reverse transcription polymerase chain reaction; SARS-CoV-2, severe acute respiratory syndrome coronavirus 2.
