# Supplementary material for: Positive association of angiotensin II receptor blockers, not angiotensin-converting enzyme inhibitors, with an increased vulnerability to SARS-CoV-2 infection in patients hospitalized for suspected COVID-19 pneumonia
Source: PLoS One. 2020 Dec 21;15(12):e0244349. doi: 10.1371/journal.pone.0244349 (PMC7751849; doi:10.1371/journal.pone.0244349)
Supplement: S3 Table — (DOC) [file pone.0244349.s003.doc]

**S3 Table. ACEIs and ARBs used in the study.**

|  | **All patients** | **COVID-19 (Group 1)** | **No COVID-19 (Group 2)** |
| --- | --- | --- | --- |
|  | **N = 684** | **N = 434** | **N = 250** |
| **ACEIs** |  |  |  |
| Benazepril | 1 (0.1) | 1 (0.2) | 0 (0.0) |
| Captopril | 2 (0.3) | 1 (0.2) | 1 (0.4) |
| Enalapril | 7 (1.0) | 3 (0.7) | 4 (1.6) |
| Lisinopril | 1 (0.1) | 0 (0.0) | 1 (0.4) |
| Perindopril | 43 (6.3) | 30 (6.9) | 13 (5.2) |
| Quinapril | 1 (0.1) | 1 (0.2) | 0 (0.0) |
| Ramipril | 35 (5.1) | 18 (4.1) | 17 (6.8) |
| Trandolapril | 2 (0.3) | 1 (0.2) | 1 (0.0) |
| All ACEIs | 92 (13.4) | 55 (12.7) | 37 (14.8) |
| **ARBs** |  |  |  |
| Candesartan | 35 (5.1) | 26 (6.0) | 9 (3.6) |
| Irbesartan | 51 (7.4) | 41 (9.4)^a^ | 10 (4.0) |
| Losartan | 7 (1.0) | 5 (1.2) | 2 (0.8) |
| Olmesartan | 1 (0.1) | 1 (0.2) | 0 (0.0) |
| Telmisartan | 11 (1.6) | 7 (1.6) | 4 (1.6) |
| Valsartan | 15 (2.2) | 10 (2.3) | 5 (2.0) |
| All ARBs | 120 (17.5) | 90 (20.7)^a^ | 30 (12.0) |

Data are number (%). ACEIs indicates angiotensin-converting enzyme inhibitors; ARBs, angiotensin II receptor blockers. One switch from perindopril to ramipril, and no switch between ARBs within the 6 months before inclusion. No switch from ACEI to ARB or from ARB to ACEI within the 6 months before inclusion.

a P < 0.05.
