# Supplementary material for: Positive association of angiotensin II receptor blockers, not angiotensin-converting enzyme inhibitors, with an increased vulnerability to SARS-CoV-2 infection in patients hospitalized for suspected COVID-19 pneumonia
Source: PLoS One. 2020 Dec 21;15(12):e0244349. doi: 10.1371/journal.pone.0244349 (PMC7751849; doi:10.1371/journal.pone.0244349)
Supplement: S4 Table — (DOC) [file pone.0244349.s004.doc]

S4 Table. Association between PCR-confirmed COVID-19 and long-term treatment with RAAS antagonists: The medium hypothesis (the 38 “probable” COVID-19 patients are excluded from analysis, which compares patients with PCR-confirmed COVID-19 and patients without COVID-19).

|  | **All patients** | **PCR-confirmed COVID-19** | **PCR COVID-19 negative** | **OR (95% CI)** | **P-value** |
| --- | --- | --- | --- | --- | --- |
|  | **N = 646** | **N = 396** | **N = 250** |  |  |
| RAAS inhibitors |  |  |  |  |  |
| ACEI | 92 (14.2) | 54 (13.6) | 38 (15.2) | 0.9 (0.6–1.4) | 0.57 |
| ARB | 109 (16.9) | 79 (19.9) | 30 (12.0) | 1.8 (1.2–2.9) | < 0.01 |
| MRB | 5 (0.8) | 5 (1.3) | 0 (0.0) | - | 0.07 |
| ≥ 1 RAAS inhibitor^a^ | 203 (31.4) | 136 (34.3) | 67 (26.8) | 1.4 (1.0–3.0) | 0.05 |

Data are number (%), unless otherwise indicated. ACEI indicates angiotensin converting enzyme inhibitor; ARB, angiotensin II type 1 receptor blocker; CI, confidence interval; COVID-19, coronavirus disease 2019; MRB, mineralocorticoid receptor blocker; OR, odds ratio; PCR, polymerase chain reaction; RAAS, renin-angiotensin-aldosterone system.

^a^ Totals are not equal to the sums of components, due to combinations of RAAS antagonists or multiple indications for RAAS antagonists.
