# Supplementary material for: Positive association of angiotensin II receptor blockers, not angiotensin-converting enzyme inhibitors, with an increased vulnerability to SARS-CoV-2 infection in patients hospitalized for suspected COVID-19 pneumonia
Source: PLoS One. 2020 Dec 21;15(12):e0244349. doi: 10.1371/journal.pone.0244349 (PMC7751849; doi:10.1371/journal.pone.0244349)
Supplement: S5 Table — (DOC) [file pone.0244349.s005.doc]

**S5 Table.** **Association between results of PCR for COVID-19 and long-term treatment with RAAS antagonists: The worst hypothesis (all “probable” COVID-19 patients are classified as no-COVID-19 patients).**

|  | **All patients** | **PCR-confirmed COVID-19** | **PCR COVID-19 negative** | **OR (95% CI)** | **P-value** |
| --- | --- | --- | --- | --- | --- |
|  | **N = 684** | **N = 396** | **N = 288** |  |  |
| RAAS inhibitors |  |  |  |  |  |
| ACEI | 93 (13.6) | 54 (13.6) | 39 (13.5) | 1.0 (0.7–1.6) | 0.97 |
| ARB | 120 (17.5) | 79 (19.9) | 41 (14.2) | 1.5 (1.0–2.3) | 0.052 |
| MRB | 6 (0.9) | 5 (1.3) | 1 (0.3) | 3.7 (0.5–27.4) | 0.20 |
| ≥ 1 RAAS inhibitor^a^ | 215 (31.4) | 136 (34.3) | 79 (27.4) | 1.4 (1.0–1.9) | 0.055 |

Data are number (%), unless otherwise indicated. ACEI indicates angiotensin converting enzyme inhibitor; ARB, angiotensin II type 1 receptor blocker; CI, confidence interval; COVID-19, coronavirus disease 2019; MRB, mineralocorticoid receptor blocker; OR, odds ratio; PCR, polymerase chain reaction; RAAS, renin-angiotensin-aldosterone system.

^a^ Totals are not equal to the sums of components, due to combinations of RAAS antagonists or multiple indications for RAAS antagonists.
