# Supplementary material for: Positive association of angiotensin II receptor blockers, not angiotensin-converting enzyme inhibitors, with an increased vulnerability to SARS-CoV-2 infection in patients hospitalized for suspected COVID-19 pneumonia
Source: PLoS One. 2020 Dec 21;15(12):e0244349. doi: 10.1371/journal.pone.0244349 (PMC7751849; doi:10.1371/journal.pone.0244349)
Supplement: Attachment — Submitted filename: PONE-D-20-22693_review.docx [file pone.0244349.s006.docx]

**Review for Manuscript #PONE-D-20-22693**

**Title: Positive association of Angiotensin II Receptor Blockers, not Angiotensin-Converting Enzyme Inhibitors, with an increased vulnerability to SARS-CoV-2 infection in patients hospitalized for suspected COVID-19 pneumonia**

Thank you for providing me the opportunity to review the paper entitled “Positive association of Angiotensin II Receptor Blockers, not Angiotensin-Converting Enzyme Inhibitors, with an increased vulnerability to SARS-CoV-2 infection in patients hospitalized for suspected COVID-19 pneumonia”.

**General comments:**

The study investigated the susceptibility of the patients who were treated with angiotensin converting enzyme inhibitors or angiotensin II receptor blockers. The authors suggested that significant association between COVID-19 pneumonia and history of taking ARBs. Although this study has remarkable contents, some questions are raised.

**Major comments:**

1. What do the authors think about the reason of discrepancy between the result of this study and previous reports regarding effects of ACEI and ARB with COVID-19 pneumonia?
2. Please describe the contents of the discussion section more concisely and conclusively.
3. As the authors have already described in the discussion section, there are similar studies already published with similar and opposite conclusions. It would be better if authors point out new academic contents of the present study in the discussion session.

**Minor comments:**

1. The authors should define which diseases were included in chronic heart disease.
2. In the text, there are two different terms were used and it can cause confusion to the readers.
1) SARSCoV-2, SARS-Cov-2, SARS-CoV-2 and SARS-COV-2
2) Angiotensin-converting enzyme type 2 and angiotensin-converting enzyme 2
3) RAAS system and RAAS
3. The authors used abbreviation before explanation of full term.
1) RNA
2) RT-PCR
3) RAAS
4) ENT
