# Supplementary material for: Positive association of angiotensin II receptor blockers, not angiotensin-converting enzyme inhibitors, with an increased vulnerability to SARS-CoV-2 infection in patients hospitalized for suspected COVID-19 pneumonia
Source: PLoS One. 2020 Dec 21;15(12):e0244349. doi: 10.1371/journal.pone.0244349 (PMC7751849; doi:10.1371/journal.pone.0244349)
Supplement: Attachment — Submitted filename: PONE-D-20-22693R1_Answers to reviewers2.docx [file pone.0244349.s008.docx]

**Reviewer #1:** The authors revised manuscript appropriately. The quality of this manuscript has improved. I suggest accept without further revision.

***Answer:*** *We thank you for your comments and the time dedicated to our manuscript.*

**Reviewer #2:** Georges and coworkers presented an interesting study: Positive association of ARB, not ACEI, with an increased vulnerability to SARS-CoV-2 infection in patients hospitalized for suspected COVID-19 pneumonia.

The authors correctly accepted suggested comments.

***Answer:*** *We thank you for your comments and the time dedicated to our manuscript.*

**Reviewer #3:** Manuscript ID: PONE-D-20-22693R1 Thank you for giving us the opportunity to review the manuscript Title: “Positive association of Angiotensin II Receptor Blockers, not Angiotensin-Converting Enzyme Inhibitors, with an increased vulnerability to SARS-CoV-2 infection in patients hospitalized for suspected COVID-19 pneumonia” A manuscript in which the author described comparative analysis suggested that ACEIs and ARBs are not similarly associated with the COVID-19 incidence, the patients with COVID-19 pneumonia receiving more frequently a previous treatment with ARBs than patients without COVID-19. We have some points we would like to refer:

Comments:

- Please add the laboratory value in baseline characteristics either correlated to covid-19 infection as CRP serum ferritin, and LDH or correlated to thromboembolic phase of the disease highly sensitive troponin and D-dimer.

***Answer:*** *Results of WBC, CRP, LDH, hs cardiac troponin T, and D dimer have been added in baseline characteristics (Table 1, pages 8-9), as suggested. Serum ferritin has not been added; ferritin was not measured in routine and was available for <10 patients only.*

- Prognostic factors like hospital stay, need for mechanical ventilation between two groups

***Answer:*** *All study patients with or without COVID-19 were hospitalized because they required oxygen supply. All patients admitted in ICU received a mechanical ventilation, and this was added in the Table 1. Hospital stay duration was also added in the Table 1.*

- Table 6 what does K means?

***Answer:*** *K indicates the constant in the regression model. In the revised version, we replaced K by “Constant”, more explicit.*
